# Organomercury oligonucleotide–polydopamine nanoparticle assemblies discriminate between target sequences by Hg(ii)-mediated base pairing†

**DOI:** 10.1039/d4ra07922a

**Published:** 2024-12-03

**Authors:** Tharun K. Kotammagari, Majid Al-Waeel, Jukka Lukkari, Tuomas Lönnberg

**Affiliations:** a Department of Chemistry, University of Turku Henrikinkatu 2 20500 Turku Finland tuanlo@utu.fi

## Abstract

A fluorescently tagged oligonucleotide hybridization probe incorporating a single 5-mercuricytosine residue was synthesized and found to adsorb on polydopamine nanoparticles much more strongly than its unmetallated counterpart. Hybridization with target sequences led to release of the probe from the nanoparticle to varying degrees depending on the nucleobase opposite to 5-mercuricytosine.

## Abstract 2

An organomercury nucleobase of a hybridization probe can engage in either adsorption on a polydopamine nanoparticle or Hg(ii)-mediated base pairing with a complementary oligonucleotide. The equilibrium is sensitive to the base pairing partner.

## Graphical abstract

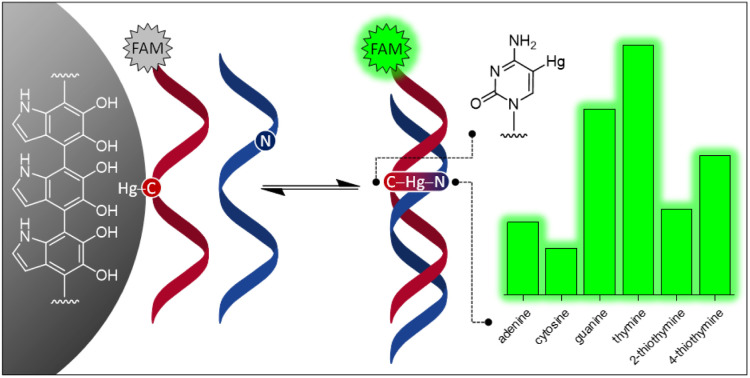

## 1. Introduction

Nanomaterials that exhibit fluorescence emitting or quenching properties are crucial for the advancement of molecular probes for the detection and investigation of nucleic acids.^1–6^ The development of this class of fluorescent sensors for assaying biomolecules has recently received considerable attention due to their inherent advantages, such as high sensitivity, operational convenience and, especially, *in situ* imaging properties. The integration of oligonucleotides with nanoparticles has emerged as a promising avenue, offering remarkable opportunities for the design of advanced sensor architectures. Several nanomaterials, such as gold nanoparticles (AuNPs),^7–9^ carbon nanotubes (CNTs),^10^ graphene oxide,^11,12^ metal–organic frameworks (MOFs),^13^ MoS_2_ nanosheets,^14^ and polydopamine nanoparticles (PDANs)^15,16^ have been utilized as quenchers in the development of fluorescent sensors.^15–17^ Although considerable progress has been made in the use of nanomaterials in nucleic acid detection, several challenges remain in controlling the assembly and fluorescence emission properties, along with issues like background interference and salt-induced aggregation, which limit detection capabilities.

Among functional nanomaterials, PDANs have gained much attention in drug delivery, therapy, and biosensing due to their biocompatibility and surface-coating properties.^18–20^ Like the majority of the nanomaterials discussed above, PDANs carry negative charges at neutral pH and, hence, repel the polyanionic phosphate backbone of DNA. The electrostatic repulsion can be alleviated by lowering the pH or masking the negative charges by increasing the electrolyte concentration. On the other hand, PDANs are capable of π–π stacking and hydrogen bonding, both of which promote adsorption.^15^ Furthermore, the catecholamine moieties of PDANs are strong ligands for various metal ions,^21–23^ allowing much more efficient adsorption through metal coordination. Such metal-mediated adsorption of DNA has been harnessed to create DNA/PDAN sensor assemblies for the detection of nucleic acids in biological media.^16^

**Scheme 1:**
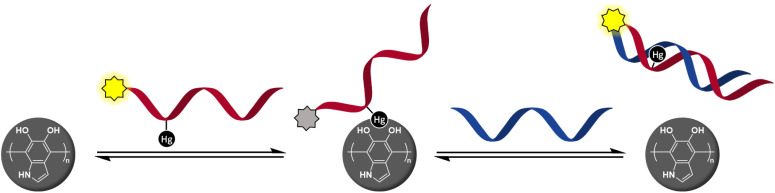
Hg(ii)-promoted adsorption of a fluorescently tagged oligonucleotide probe quenches emission and hybridization with a target sequence restores it.

Our previous studies on organomercury oligonucleotides as hybridization probes^24–28^ have revealed an increased hybridization affinity and in some cases robust discrimination of all the canonical bases, 2-thiothymine and 4-thiothymine through metal-mediated base pairing.^29–34^ The state-of-the-art methods for the identification of single-nucleotide polymorphisms (SNPs) require as many as four different hybridization probes interrogating the polymorphic base through Watson–Crick base pairing.^35,36^ In contrast, we have been able to obtain clearly different UV melting temperatures^24,26,28^ or fluorescence signals^25^ for all canonical nucleobases using only a single organomercury hybridization probe. After learning of the success of metal-mediated adsorption in developing DNA/PDAN sensors,^16^ it occurred to us that the covalently bound Hg(ii) ions of organomercury oligonucleotides^37^ might also exhibit sufficient affinity to the catecholamine ligands on the surface of PDANs to promote adsorption (Scheme 1). On hybridization of the organomercury oligonucleotide with a complementary sequence, the Hg(ii) ion would become engaged in Hg(ii)-mediated base pairing and thus be no longer available for catecholamine coordination, leading to dissociation of the resulting duplex from the PDAN. As an important advantage over the previously reported DNA/PDAN sensors based on coordinative interactions with metal ions, the organomercury oligonucleotide remains undissociated even at high dilution in aqueous media, as exemplified by numerous prior studies.^37,38^ The present article describes the preparation and testing of a novel sensor based on this reasoning.

## 2. Results and discussion

### 2.1 Synthesis of the polydopamine nanoparticles

The PDANs were synthesized according to a well-established literature procedure, which involves an oxidative self-polymerization of dopamine.^39^ The size of the PDANs was determined by dynamic light scattering (DLS) and transmission electron microscopy (TEM) (Fig. S5 and S6 in the ESI†). An average diameter of approximately 170 nm was obtained by both methods.

### 2.2 Oligonucleotide synthesis

**Scheme 2:**
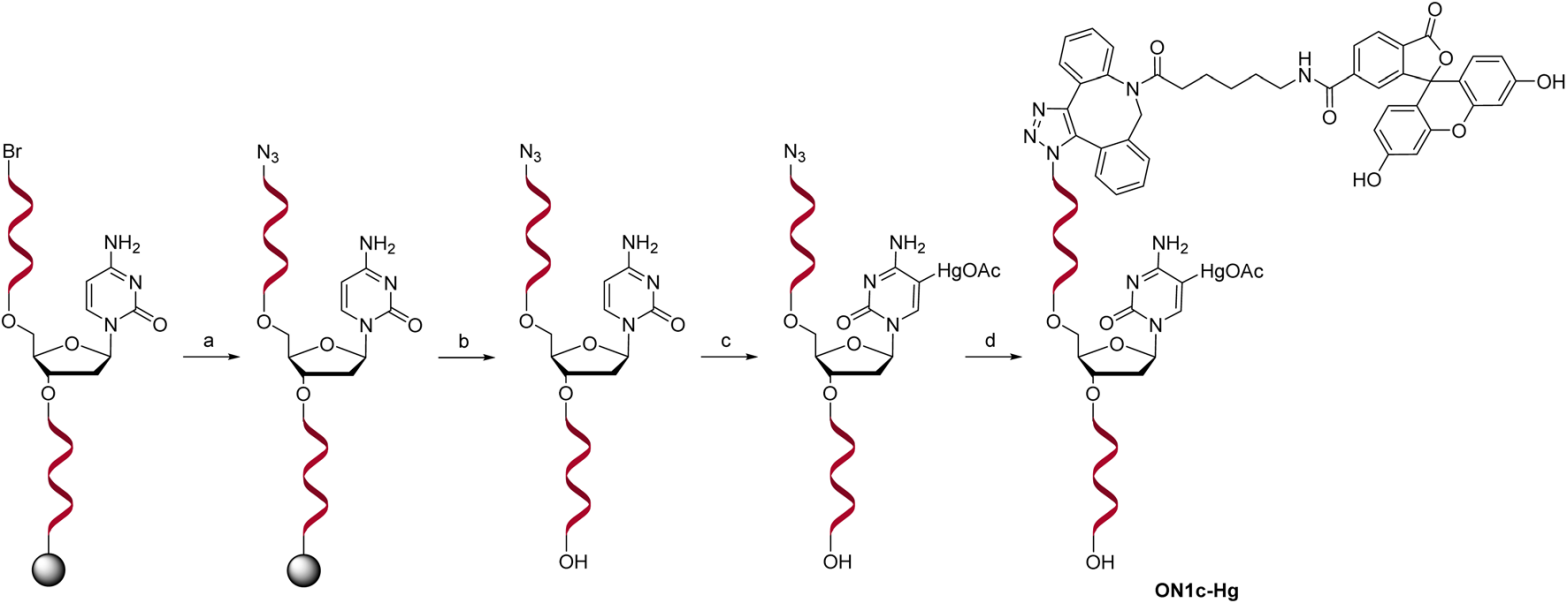
Post-synthetic transformations to afford the mercurate probe oligonucleotide ON1c-Hg. Reagents and conditions: (a) TMGA, NaI, DMF, 60 °C, 90 min; (b) NH_3_, H_2_O, 55 °C, 16 h; (c) Hg(OAc)_2_, H_2_O, 55 °C, (d) 48 h; 6-FAM-DBCO, DMF, 25 °C, 16 h.

For the sensing component, oligonucleotide ON1c-Hg, bearing a fluorescent tag and a single 5-mercuricytosine residue, was synthesized. The mercurated nucleobase was positioned seven nucleotides away from the 5′-terminal fluorophore, sufficiently far to prevent direct interaction, but at the same time, sufficiently near so that Hg(ii)-mediated adsorption on the PDAN would cause a quenching of the fluorescence. The 24-mer sequence (Table 1) of ON1c-Hg was first assembled by the conventional phosphoramidite strategy on an automated synthesizer. A 6-bromohexyl group^25^ was introduced at the 5′-end for subsequent coupling of the fluorophore. To prevent unwanted mercuration, all cytosine building blocks were replaced with 5-methylcytosines, except for the single residue designated for mercuration. Additionally, the order of the post-synthetic transformations had to be planned carefully to prevent mercuration of the FAM-6 fluorophore. Accordingly, after the synthesis of the 5′-bromo oligonucleotide sequence, the 5′-bromide was displaced by an azide group by treatment with tetramethylguanidinium azide (TMGA) on solid support (Scheme 2). The resulting 5′-azide oligonucleotide intermediate was then cleaved from the solid support with aqueous ammonia and purified by RP-HPLC.

**Table 1:** Sequences of the oligonucleotides used in this study

Oligonucleotide	Sequence (5′–3′)^a^
ON1c	6-FAM-AC^m^G C^m^AT C̲TG TGA AGA GAA C^m^C^m^T GGG
ON1c-Hg	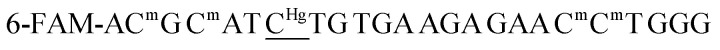
ON2a	CCC AGG TTC TCT TCA CAA̲ ATG CGT
ON2c	CCC AGG TTC TCT TCA CAC̲ ATG CGT
ON2g	CCC AGG TTC TCT TCA CAG̲ ATG CGT
ON2t	CCC AGG TTC TCT TCA CAT̲ ATG CGT
ON2s^2^t	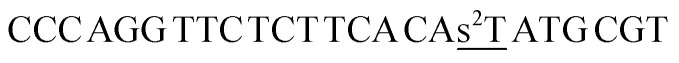
ON2s^4^t	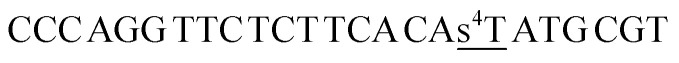

a C^m^ refers to 5-methylcytosine, C^Hg^ to 5-mercuricytosine, s^2^T to 2-thiothymine and s^4^T to 4- thiothymine. In each sequence, the residue varied in the hybridization experiments has been underlined.

Mercuration of the azide oligonucleotide intermediate was accomplished by treatment with aqueous mercuric acetate at 55 °C for 48 h. The product mixture was fractioned by RP-HPLC and identity of the desired oligonucleotide intermediate was confirmed by mass spectrometry. Finally, the fluorophore was introduced by strain-promoted azide–alkyne cycloaddition (SPAAC) of the 6-isomer of FAM-DBCO with the 5′-azide-functionalized oligonucleotide. The unmercurated reference oligonucleotide ON1c was synthesized in the same way except that the mercuration step was omitted. The complementary oligonucleotides ON2a, ON2c, ON2g, ON2t, ON2s^2^t and ON2s^4^t (Table 1), in turn, were synthesized from commercially available phosphoramidite building blocks following recommended procedures. The final products were purified by RP-HPLC (Fig. S1 and S2 in the ESI†), characterized mass spectrometrically (Fig. S3 and S4 in the ESI†) and quantified UV spectrophotometrically.

### 2.3 Affinity of the oligonucleotide hybridization probes for the polydopamine nanoparticles

**Fig. 1:**
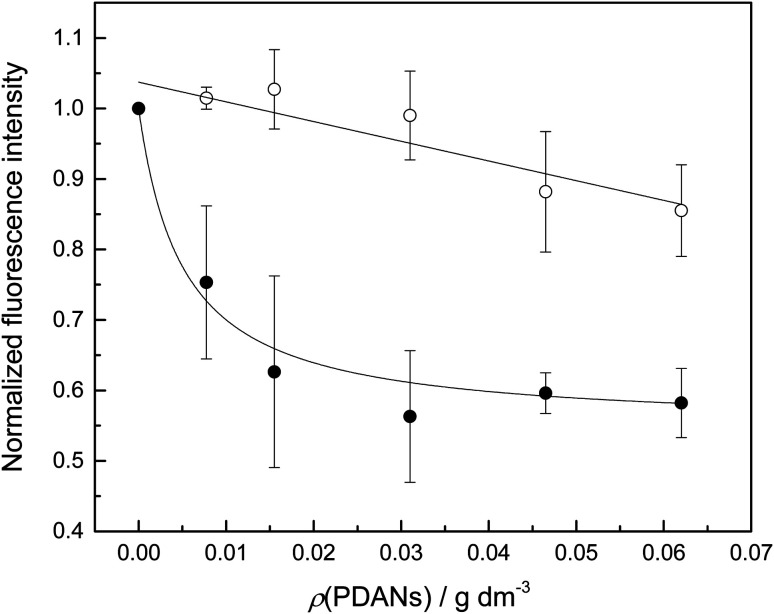
Fluorescence emission of oligonucleotides ON1c (○) and ON1c-Hg (●) as a function of PDAN concentration; *T* = 25 °C; pH = 7.4 (20 mM cacodylate buffer); *I*(NaClO_4_) = 0.10 M; [oligonucleotides] = 10 nM; *λ*_ex_ = 493 nm; *λ*_em_ = 517 nm.

While inorganic Hg(ii) shows high affinity for polydopamine,^40^ it was not certain that a single 5-mercuricytosine residue would suffice to bind the oligonucleotide probe to the PDAN under conditions in which the unmetallated nucleic acids remain unbound. Therefore, we compared the PDAN-binding affinity of the mercurated oligonucleotide ON1c-Hg and its unmercurated counterpart ON1c by measuring their fluorescence emission at increasing PDAN concentrations. As anticipated, both oligonucleotides exhibited high fluorescence emission at 517 nm in the absence of PDANs. The fluorescence intensity decreased on increasing PDAN concentration but the profiles obtained for ON1c-Hg and ON1c (Fig. 1) were very different. With ON1c, the decrease was linear, suggesting a low binding affinity. Almost 90% of the emission persisted even at the highest PDAN concentration used. In contrast, with ON1c-Hg, fluorescence emission initially decreased steeply with increasing PDAN concentration before reaching a plateau. Assuming that the number of binding sites on the surface of the PDANs greatly exceeds the number of mercurated oligonucleotides in the sample, the dependence of fluorescence intensity on PDAN concentration can be expressed by eqn (1).1
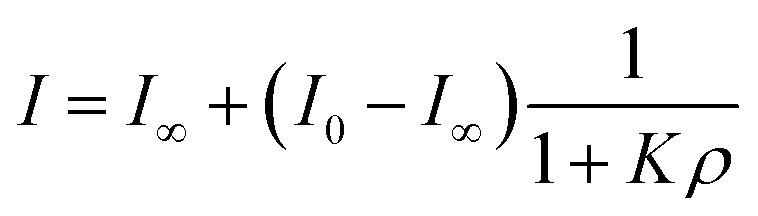


*I*_0_ and *I*_∞_ are fluorescence intensities in the absence and at an infinite concentration of PDANs, *K* is the adsorption affinity constant and *ρ* the mass concentration of the PDANs. Values of *I*_∞_ = 0.55 ± 0.02 and *K* = 200 ± 70 g^−1^ dm^3^ were obtained by non-linear least-squares fitting, whereas *I*_0_ was defined as = 1. The residual emission at high PDAN concentration probably stems from flexibility of the single-stranded oligonucleotide, allowing the fluorophore to spend some time away from the PDAN even when the oligonucleotide is adsorbed. Incomplete adsorption of ON1c-Hg appears a less likely explanation given the observed saturation at high PDAN concentration. The relatively high affinity of the mercurated probe ON1c-Hg (compared to its metal-free counterpart) for the PDANs, probably attributable to mercury coordination by the catechol moieties, was encouraging from the point of view of the intended sensor application. In contrast, the low affinity of the unmercurated probe ON1c precludes a meaningful analysis.

### 2.4 Sequence selectivity of the polydopamine nanoparticle—oligonucleotide assemblies

For assessing the potential of the PDAN—ON1c-Hg assembly as a sensor, the PDAN concentration was fixed at the value where the difference in the fluorescence intensity of ON1c and ON1c-Hg peaked (0.016 mg dm^−3^), while keeping the other parameters the same as in the affinity measurements. ON1c-Hg and the PDANs were first incubated for approximately 10 min, after which an equimolar amount of one of the complementary oligonucleotides ON2a, ON2c, ON2g, ON2t, ON2s^2^t and ON2s^4^t was added. Hybridization of ON1c-Hg with these oligonucleotides places each of the canonical nucleobases, as well as 2- and 4-thiothymine, opposite to the 5-mercuricytosine residue. With ON2a, fluorescence emission was recorded 10 min, 20 min and 24 h after the addition and found to be unchanged. All subsequent measurements were performed 10 min after addition of the complementary oligonucleotide. Recovery of the fluorescence, expressed by eqn (2), was observed in each case, indicating duplex formation and subsequent dissociation from the PDAN.2
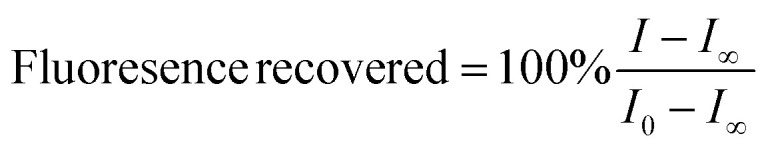


**Fig. 2:**
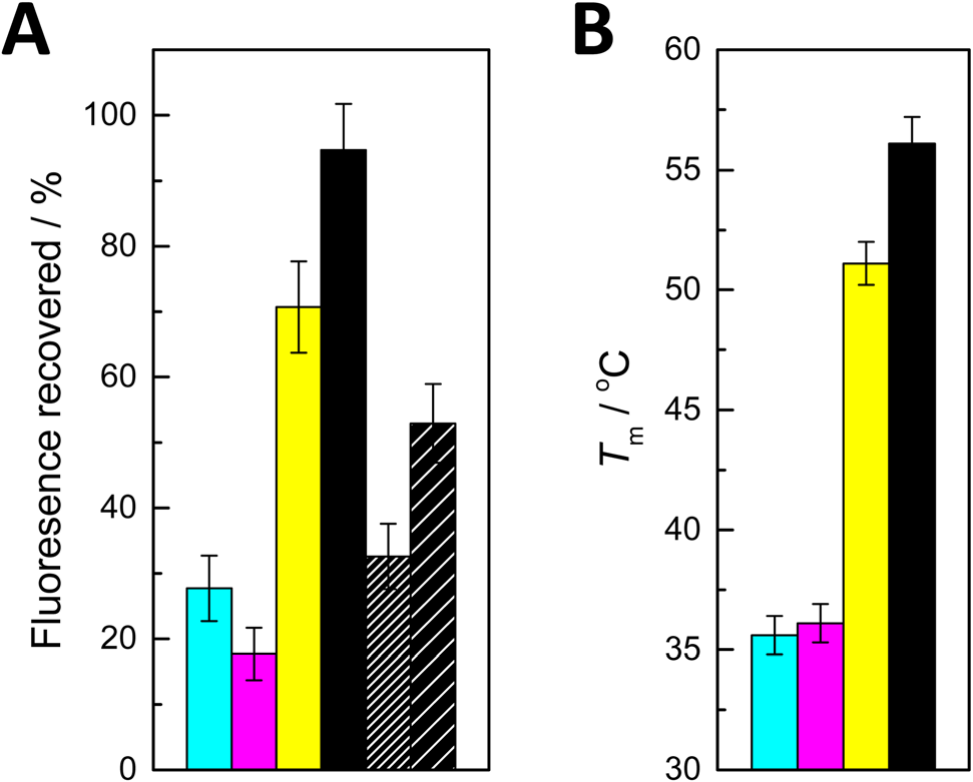
(A) Recovery of fluorescence on addition of ON2a (cyan), ON2c (magenta), ON2g (yellow), ON2t (black), ON2s^2^t (black with dense hash) and ON2s^4^t (black with sparse hash) to PDAN-bound ON1c-Hg; *T* = 25 °C; pH = 7.4 (20 mM cacodylate buffer); *I*(NaClO_4_) = 0.10 M; [oligonucleotides] = 10 nM; *ρ*(PDAN) = 0.016 mg dm^−3^; *λ*_ex_ = 493 nm; *λ*_em_ = 517 nm. (B) Previously reported^27^ UV melting temperatures of double-helical 11-mer DNA oligonucleotides incorporating a Hg(ii)-mediated base pair between 5-mercuricytosine and adenine (cyan), cytosine (magenta), guanine (yellow) and thymine (black).

*I* is the fluorescence intensity recorded after addition of the complementary oligonucleotide and *I*_0_ and *I*_∞_ are the limits determined during the affinity measurements. The values obtained are summarized in Fig. 2A.

The effects of adding the oligonucleotides consisting solely of canonical nucleobases fell into two distinct categories. With ON2a and ON2c, recovery of fluorescence was modest (28 and 18%, respectively). In contrast, addition of ON2g and ON2t led to 71 and 95% recovery of the fluorescence, respectively. These results are in good agreement with the previously reported^27^ thermal stabilities of corresponding short duplexes (Fig. 2B) and with the tendency of Hg(ii) to displace a proton from the N1 of guanine and the N3 of thymine upon coordination. The differences in fluorescence recovery were sufficiently large for a robust discrimination between most of the canonical nucleobases. The sole exception was differentiating adenine from cytosine, which could not be achieved reliably with the present system.

Curiously, when either of the thiopyrimidine sequences ON2s^2^t and ON2s^4^t was added, the fluorescence recovery was lower compared to ON2t and ON2g, but higher compared to ON2a and ON2c. This result may seem unexpected given the high stabilities previously reported for duplexes involving Hg(ii)-mediated base pairing with 2- and 4-thiothymine.^28^ It should be pointed out, however, that in those cases the Hg(ii) ion was offset from the centerline of the base pair, allowing it to coordinate to an exocyclic thio substituent with only minor distortion of the geometry. In the present case, coordination of the Hg(ii) to an exocyclic thio substituent would lead to a considerable shear with either isomer of thiothymine.

## 3. Conclusion

In summary, a single 5-mercuricytosine residue allows efficient adsorption of oligonucleotide hybridization probes on PDANs under conditions where respective metal-free oligonucleotides remain largely unabsorbed, in all likelihood through coordination of the Hg(ii) ion to a catecholamine moiety. Addition of a complementary target oligonucleotide leads to partial dissociation of the probe and the equilibrium reflects differences in the strength of Hg(ii)-mediated base pairing between 5-mercuricytosine and the nucleobase opposite to it within the target sequence. These results demonstrate the potential of organometallic oligonucleotide—PDAN assemblies as novel sensors for SNP genotyping. The advantages of our method include a simple and rapid procedure and the utilization of affordable and biodegradable nanoparticles.

## 4. Experimental section

### 4.1 General experimental methods

HPLC elution buffers (pH 7.0) were prepared using freshly distilled analytical grade triethylamine (Et_3_N). All other reagents and solvents were obtained commercially and used without further purification. Mass spectra were recorded using a Waters ACQUITY RDa mass spectrometer, UV spectra on a Jenway Genova Nano microvolume UV/vis spectrophotometer and dynamic light scattering (DLS) spectra on a Zetasizer Nano 90 (Malvern) instrument. Transmission electron microscopy (TEM) images were captured with a JEM-1400 Plus TEM instrument. Fluorescence measurements were performed on an FLS 1000 (Edinburgh instruments) fluorescence spectrometer. X-ray photoelectron spectroscopy (XPS) measurements were conducted using a Nexsa XPS instrument (Thermo Scientific). Fourier-transform infrared spectroscopy (FTIR) was carried out using a Vertex 70 instrument (Bruker).

### 4.2 Oligonucleotide synthesis

Oligonucleotides ON1c, ON1c-Hg, ON2a, ON2c, ON2g, ON2t, ON2s^2^t and ON2s^4^t were synthesized using an ÄKTA oligopilot plus 10 DNA/RNA synthesizer on a controlled pore glass (CPG) support, employing the conventional phosphoramidite method with 5-(benzylthio)-1*H*-tetrazole as the activator and the previously reported^25^ 6-bromohexyl phosphoramidite building block for introduction of the 5′-bromo group. The trityl response indicated that all coupling reactions achieved near-quantitative efficiency. Following synthesis, the support-bound oligonucleotides ON1c, ON1c-Hg, ON2a, ON2c, ON2g, ON2t and ON2s^2^t were treated with 25% aqueous NH_3_ at 55 °C for 16 h to cleave the linker and to remove the phosphate and base protecting groups. With ON2s^4^t, the cyanoethyl protecting groups of the phosphate linkages were removed first by treating with 1.0 M DBU in anhydrous acetonitrile at 25 °C for 2 h. This treatment was followed by full deprotection using 50 mM NaSH in 25% aqueous NH_3_ at 25 °C for 24 h. The manufacturer of the 4-thiothymidine phosphoramidite building block recommends this protocol to prevent ammonolysis of the S-cyanoethyl group. The crude oligonucleotides were purified by reverse-phase high-performance liquid chromatography (RP-HPLC) employing a Hypersil ODS C18 column (250 × 4.6 mm, 5 μm) and a BioZen™ Oligo column (150 × 4.6 mm, 2.6 μm). A linear gradient (5–45% over 20 min) of MeCN in 50 mM aqueous triethylammonium acetate was employed, with flow rates of 1.0 mL min^−1^ for the Hypersil ODS C18 column and 0.6 mL min^−1^ for the BioZen Oligo column.

For introduction of the fluorescent tag to ON1c-Hg and ON1c, the 5′-bromo group was converted to an azido group. This conversion was performed on solid support within the synthesis column by adding tetramethylguanidinium azide (TMGA) (32 mg, 100 eq.) and NaI (30 mg, 100 eq.) in dry DMF (2 mL), followed by heating the mixture at 60 °C for 90 min. The reaction mixture was periodically agitated by manually transferring the solution between two syringes. Subsequently, the support-bound oligonucleotides were washed sequentially with DMF, DCM, H_2_O and MeOH and dried under vacuum. Finally, the oligonucleotides were cleaved from the solid support and purified by RP-HPLC on a Hypersil ODS C18 column.

A portion (30 nmol) of the 5′-azido oligonucleotide was incubated with Hg(OAc)_2_ (10 μmol) in 100 μL of H_2_O at 55 °C for 48 h. The resulting mercurated oligonucleotide was purified by RP-HPLC on a Hypersil ODS C18 column. Subsequently, the mercurated 5′-azido oligonucleotide was coupled to fluorescein-DBCO by strain-promoted alkyne–azide cycloaddition (SPAAC). For this, FAM-DBCO (13.5 mg) was dissolved in DMF (200 μL) and two equivalents (0.3 μL) were added to an aqueous solution of the oligonucleotide (15 nmol). The reaction mixture was vortexed at room temperature for 16 h, followed by RP-HPLC purification on a BioZen™ Oligo column (Fig. S2 in the ESI†). The rest of the 5′-azido oligonucleotide (30 nmol) was conjugated with fluorescein-DBCO and the crude product purified under the same conditions (Fig. S1 in the ESI†). Finally, both of the purified products (ON1c and ON1c-Hg) were characterized by mass spectrometry (Fig. S3 and S4 in the ESI†) and quantified by UV spectrophotometry.

### 4.3 PDAN synthesis and characterization

A solution containing 200 mg of dopamine in 100 mL of freshly prepared 8.5 mM aqueous NaOH was vigorously stirred at 50 °C in a well-ventilated fume hood. After 5 h reaction, the resulting black suspension was cooled and centrifuged at 9300 rpm for 20 min. The supernatant was removed and the pellet resuspended in H_2_O. The washing and centrifugation procedure was repeated four times, after which the PDANs were redispersed in H_2_O for subsequent experiments. The dynamic size and morphology of the PDANs were determined by DLS Zetasizer Nano 90 (Malvern) and TEM, respectively (Fig. S5 and S6 in the ESI†). Additionally, the synthesized PDANs were subjected to characterization *via* FTIR and XPS (Fig. S7 and S8 in the ESI†).

### 4.4 Fluorescence measurements

Fluorescence emission spectra were obtained over a range of 500–700 nm using an excitation wavelength of 493 nm and a dwell time of 0.2 seconds per data point. The samples consisted of 10 nM probe oligonucleotide ON1c or ON1c-Hg, 20 mM cacodylate buffer, maintained at pH 7.4 with an ionic strength of 0.10 M (adjusted with NaClO_4_). With the mercurated probe ON1c-Hg, emission spectra were recorded both before and after addition of a 10 nM concentration of each one of the complementary strands ON2a, ON2c, ON2g, ON2t, ON2s^2^t and ON2s^4^t.

## Data availability

The data supporting this article have been included as part of the ESI.†

## Author contribution

T. K. K.: conceptualization, data curation, formal analysis, funding acquisition, investigation, methodology, validation, visualization, writing – original draft, writing – review and editing; M. A.-W.: investigation, methodology, writing – review and editing; J. L.: methodology, supervision, writing – review and editing; T. L.: data curation, formal analysis, project administration, supervision, visualization, writing – review and editing.

## Conflicts of interest

The authors report no conflict of interest.

## Supplementary Material

RA-014-D4RA07922A-s001
