# Supplementary material for: Organomercury oligonucleotide–polydopamine nanoparticle assemblies discriminate between target sequences by Hg(ii)-mediated base pairing
Source: RSC Adv. 2024 Dec 3;14(51):38279–84. doi: 10.1039/d4ra07922a (PMC11612767; doi:10.1039/d4ra07922a)
Supplement: RA-014-D4RA07922A-s001 [file RA-014-D4RA07922A-s001.pdf]

Supplementary Information (SI) for RSC Advances.
This journal is © The Royal Society of Chemistry 2024

Contents

Figure S1. RP-HPLC traces of A) crude and B) purified ON1c. S2

Figure S2. RP-HPLC traces of A) crude and B) purified ON1c-Hg. S3

Figure S3. Mass spectrum of ON1c. S4

Figure S4. Mass spectrum of ON1c-Hg. S5

Figure S5. DLS spectrum of the polydopamine nanoparticles. S6

Figure S6. TEM micrographs of the polydopamine nanoparticles. S6

Figure S7. FTIR spectra of dopamine and polydopamine nanoparticles. S7

Figure S8. A) Survey XPS spectrum and high-resolution XPS spectra of B) O1s, C) N1s and D) C1s orbitals of the polydopamine nanoparticles. S8

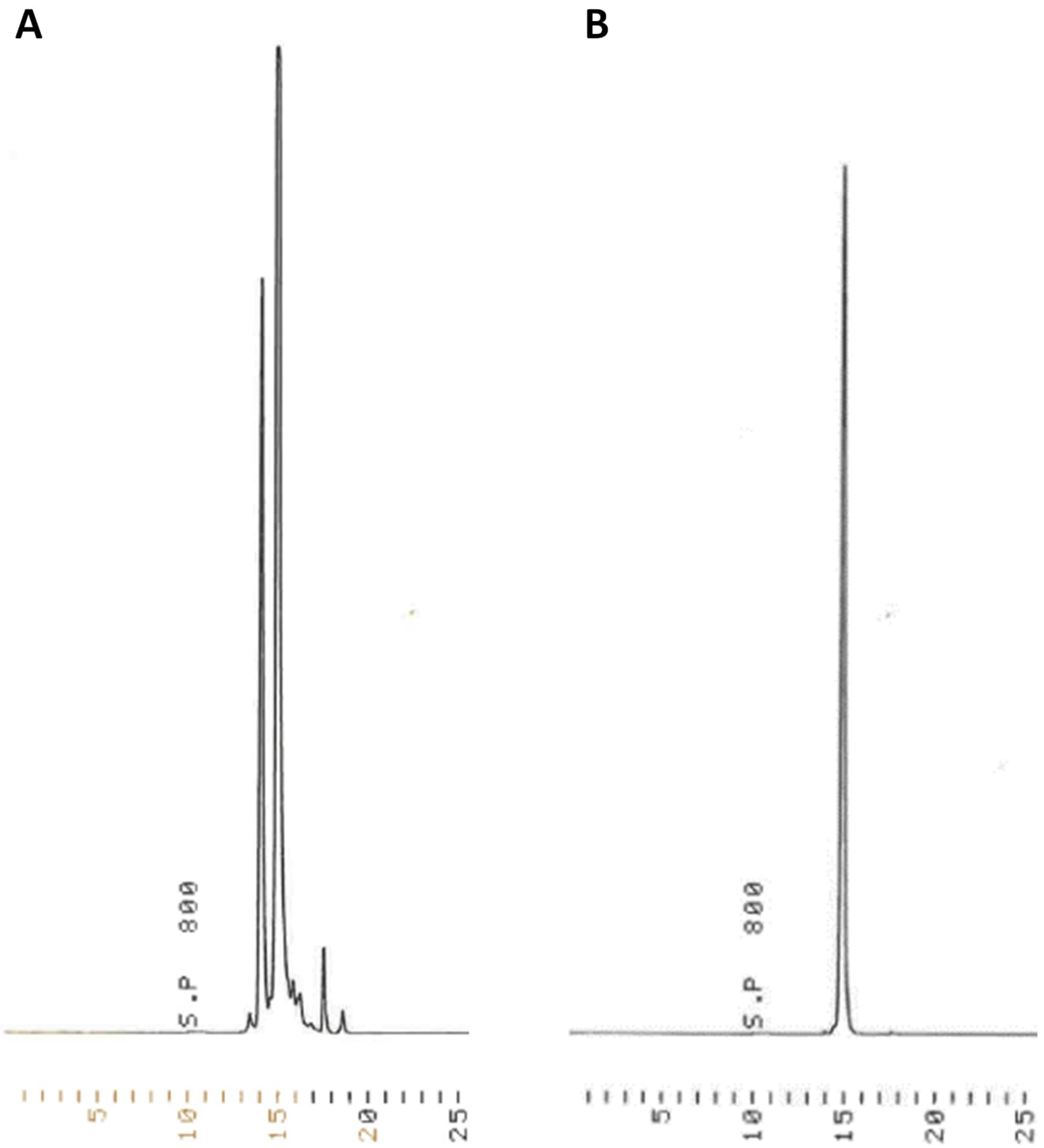

Figure S1. RP-HPLC traces of A) crude and B) purified ON1c; BioZen™ Oligo column (150 × 4.6 mm, 2.6 µm); linear gradient (5—45 % over 20 min) of MeCN in 50 mM aqueous triethylammonium acetate; flow rate = 0.6 mL min^{-1}; λ = 260 nm.

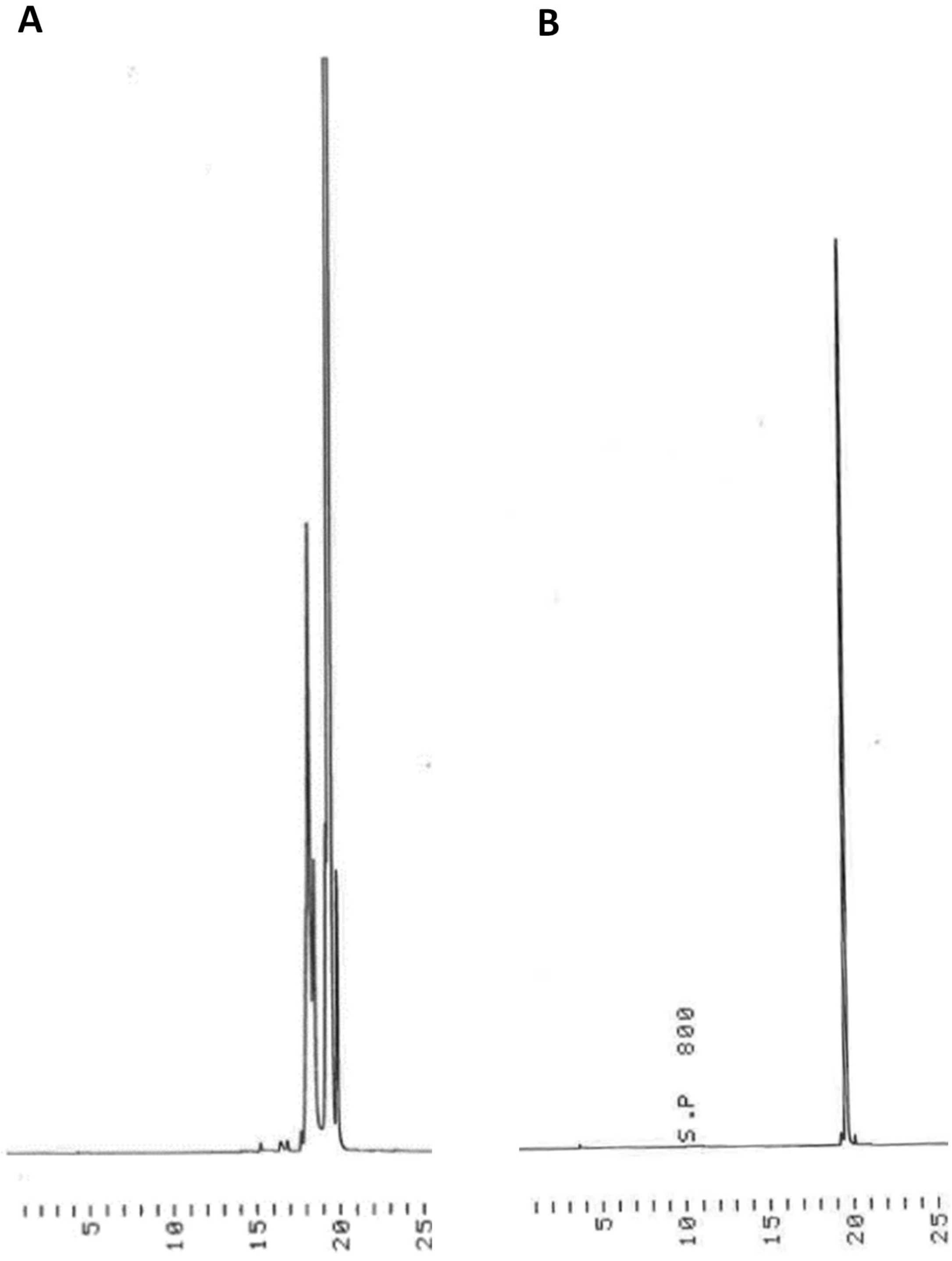

Figure S2. RP-HPLC traces of A) crude and B) purified ON1c-Hg; BioZen™ Oligo column (150 × 4.6 mm, 2.6 µm); linear gradient (5—45 % over 20 min) of MeCN in 50 mM aqueous triethylammonium acetate; flow rate = 0.6 mL min^{-1}; λ = 260 nm.

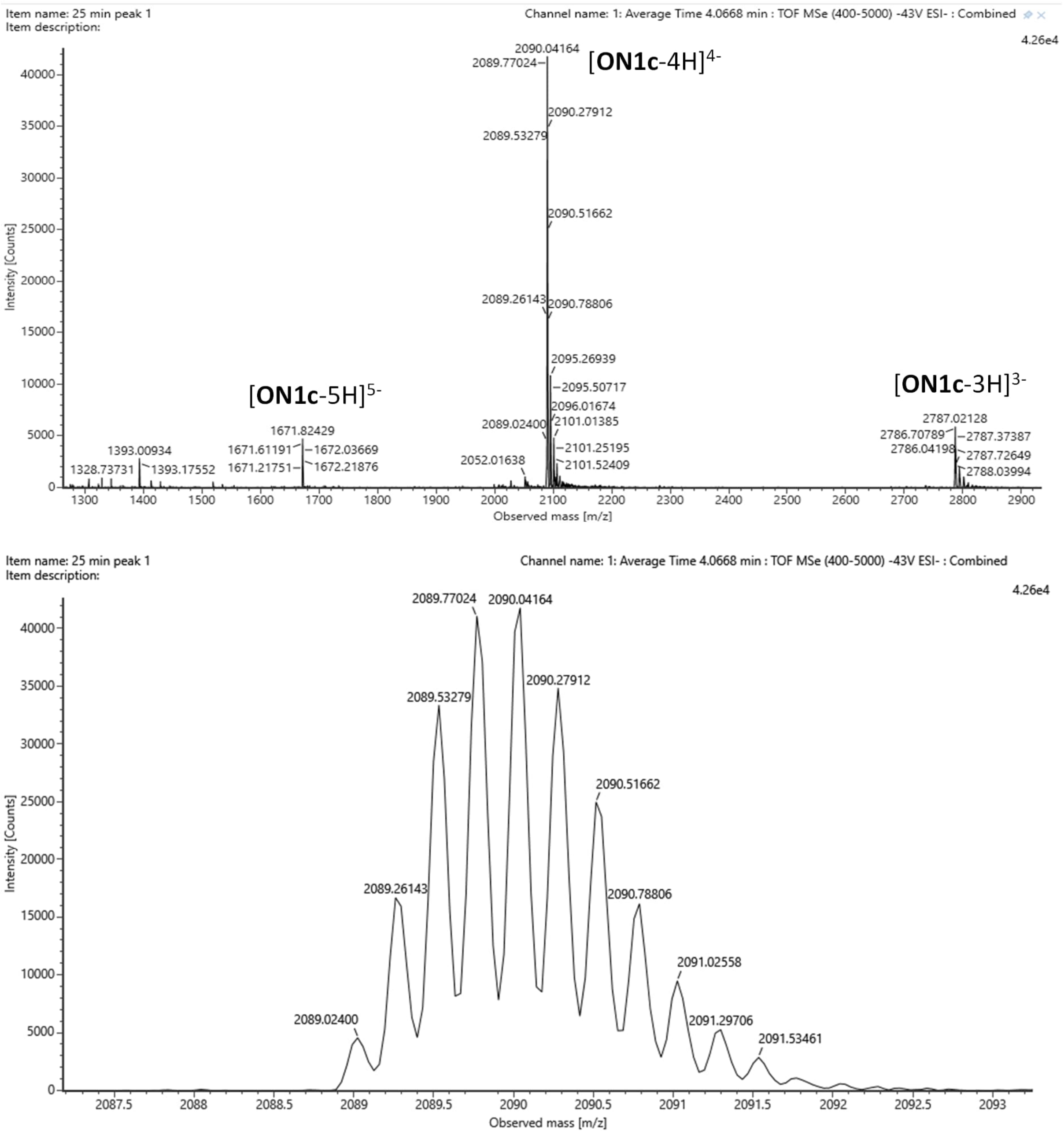

Figure S3. Mass spectrum of ON1c.

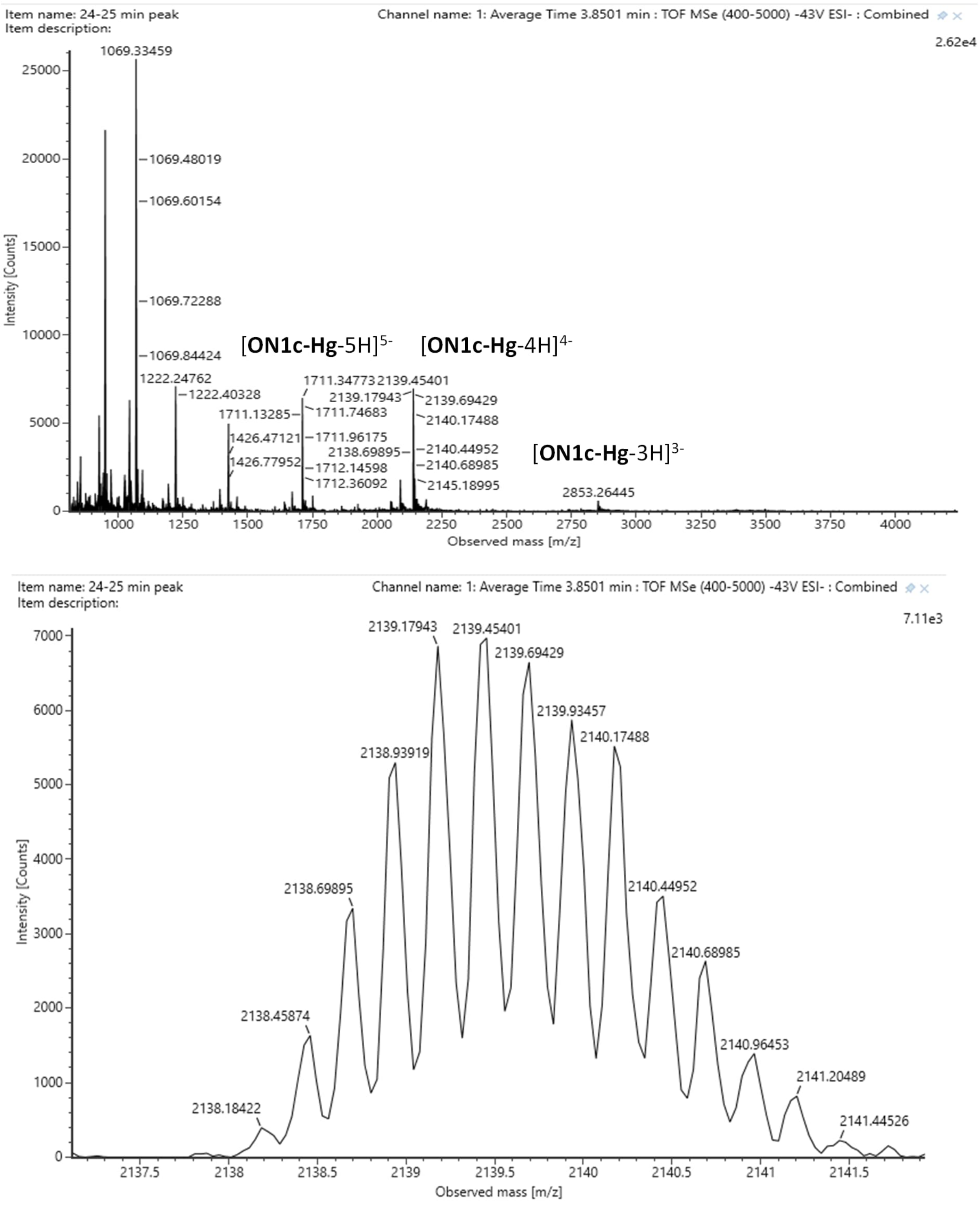

Figure S4. Mass spectrum of ON1c-Hg.

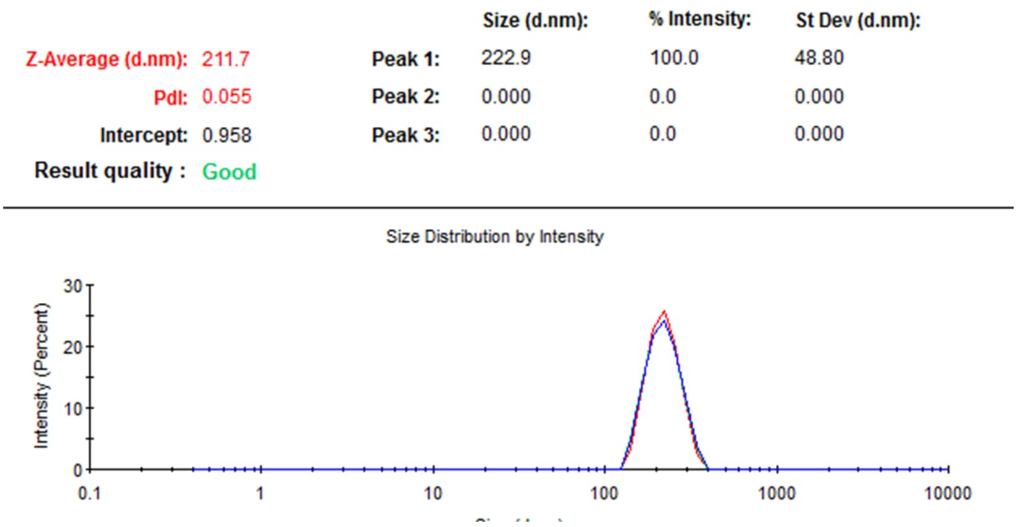

Figure S5. DLS spectrum of the polydopamine nanoparticles.

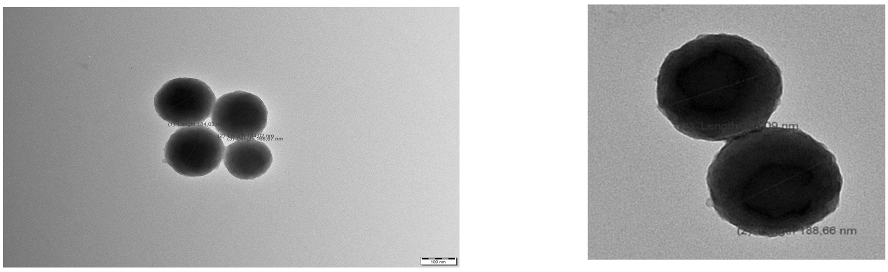

Figure S6. TEM micrographs of the polydopamine nanoparticles.

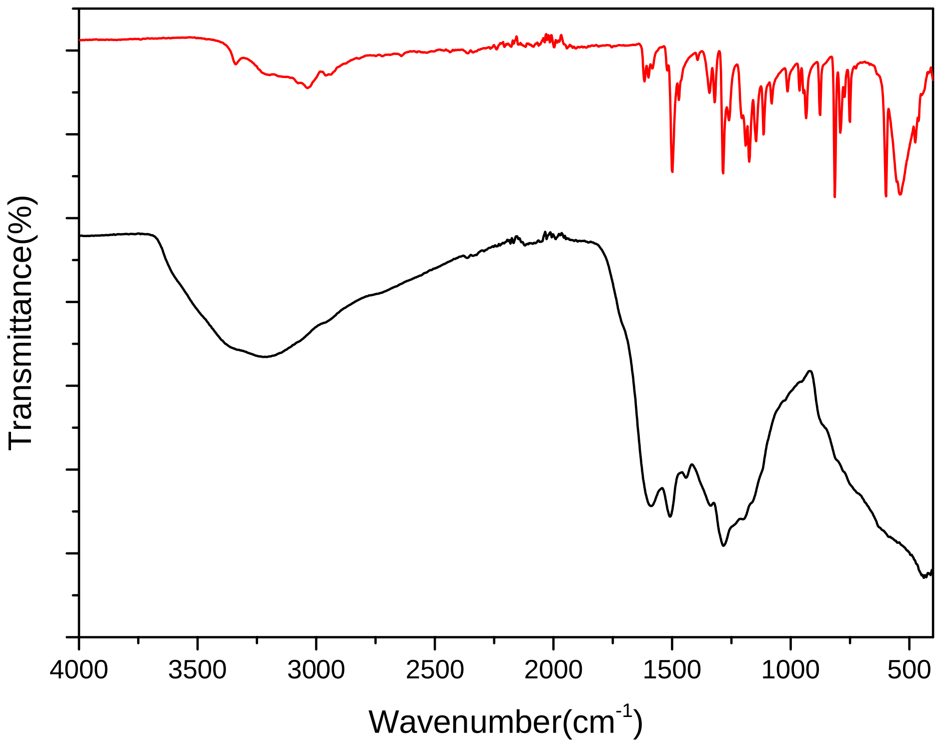

Figure S7. FTIR spectra of dopamine (red line) and polydopamine nanoparticles (black line).

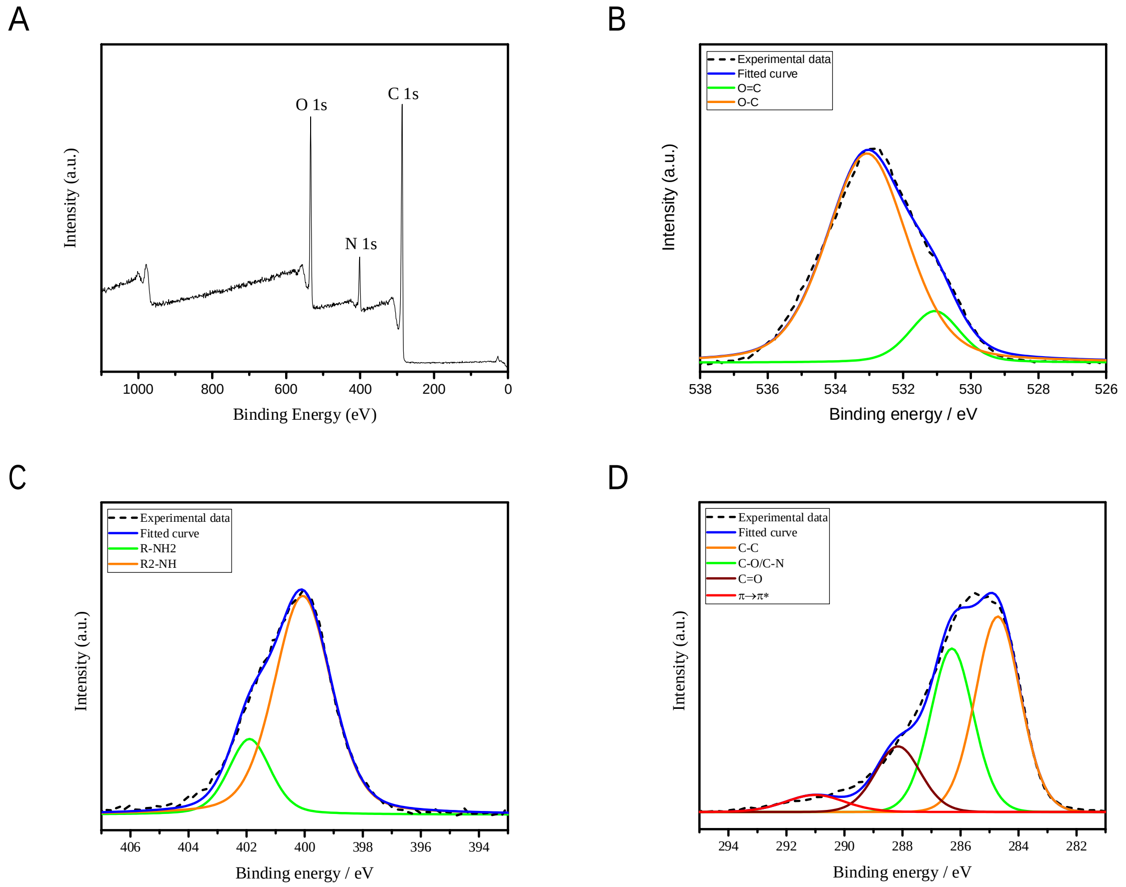

Figure S8. A) Survey XPS spectrum of the polydopamine nanoparticles and high-resolution XPS spectra of the B) O 1s, C) N 1s and D) C 1s regions.
